# Factors affecting digital technology access in vocational education

**DOI:** 10.1038/s41598-023-32755-6

**Published:** 2023-04-07

**Authors:** Akhmad Habibi, Sofyan Sofyan, Amirul Mukminin

**Affiliations:** Fakultas Keguruan dan Ilmu Pendidikan, Universitas Jambi, Jambi, Indonesia

**Keywords:** Psychology, Human behaviour

## Abstract

If policies are not thoroughly designed, technology integration may fail. As a result, users’ perceptions of technology, especially access to digital technology, are critical for technology integration in education. This study aimed to develop and validate a scale to model factors affecting digital technology access for instructional use in Indonesian vocational schools. The study also reports the structural model of the path analysis and tests of differences based on geographical areas. A scale adapted from prior studies was established, validated, and examined for its validity and reliability. A total of 1355 responses were measurable; partial least squares structural equation modeling (PLS-SEM) and t-test procedures were applied for the data analysis. The findings informed that the scale was valid and reliable. For the structural model, the strongest relationship emerged between motivational access and skills access, while the lowest existed between material access and skills access. However, motivational access has an insignificant effect on instructional use. The t-test results show that geographical areas were significantly different regarding all involved variables.

## Introduction

Digital technology has been very important in today’s modern civilization as a source of communication, entertainment, information, and education^1^. Nevertheless, due to the digital divide, not everyone has equal access to technology. The digital divide measures the gap between people who might have access to technology and those who do not, which amplifies related disparities such as financial, informational, social, and educational divides. In the past, the issue focused solely on physical access to digital technology, such as computers and the Internet^2,3^. However, physical access may not be the only factor; other characteristics, such as motivational and skills access, should also be addressed^2^. Differences in personal and social status result in an unequal distribution of resources in the community, unbalanced access to digital technologies, and social participation. As a result, all societies must investigate the mitigation of the digital divide.

Access to technology was already addressed in academic contexts. This has, nevertheless, been dealt within a limited manner^4^; for instance, it mainly concentrated on students’ physical access, whereas it solely focused on verifying and applying an instrument for measuring technology access in learning^5^. Limited studies on technological access among teachers have been published, especially in specific contexts and settings^8,67^. For instance, Moldovan et al. (2022) informed perspectives from 10 mathematic teachers on the digital divide during Covid-19 teaching, elaborating on the importance of an in-depth understanding of technology-associated systemic inequalities in marginalized urban communities and strategies to integrate technology in urban areas^7^.

In the vocational school teachers’ context, which is the focus of the current study, more limited studies were conducted^8^. Vocational education is a type of education that prepares students to be employed or self-employed with requisite skills, preparing individuals to work as a technician or to take up employment in a skilled craft. In Indonesia, vocational education has nine areas of expertise, from technology and engineering to creative industries^9^. Teachers have significant roles in shaping how technology is integrated during teaching^6,10^. Therefore, the current study contributes to filling the gap by aiming to report the scale validity for a model that involves factors affecting digital technology access in a vocational context of a developing country setting, Indonesia. The model was evaluated through PLS-SEM procedures to test the structural hypotheses. Besides, a test of differences was also addressed based on the participants’ geographical areas for all involved variables.

## Literature review

Van Dijk’s theory has established the rectification of access to technology conceptions^2,3^. The theory promoted the rule of technological access called successive technology dimensional norms by breaking the thought into four parts of access (motivational, material, skills, and usage)^2,3^. Technical access challenges shifted from motivational and material access (1st two phases) to skills and usage (2nd two phases)^2^. The digital divide might occur at any time or even at all stages. The process of using digital technology has indeed been characterized as access to digital technology^11^. At first, the approach focused on attitude and motivation before moving on to material or physical access. The theory progressed from material access to skills and utilization^12,13^.

### The use of digital technologies in vocational schools

Vocational school teachers’ comprehension of how knowledge can be developed and how technology-related competencies can be improved through various tools^14^. After school closure due to the Covid-19 pandemic, the use of digital technology in schools is significantly implemented and becomes a trend in education, including vocational education^15^. The demands for teachers to use digital technology during teaching are certainly expanding; thus, they need to improve their knowledge and competencies in digital technology use for instruction. Using digital technology in teaching can improve skills for vocational school teachers that can make their students more competent and skillful as future generations for better workforces^14–16^. However, barriers to digital technology use during teaching can hinder the teachers from using technology during teaching^14–17^. In the context of vocational education, some reports informed barriers to digital technologies used faced by teachers, namely teachers’ lack of confidence, competence, and access to digital technology resources^17,18^. Other studies revealed that lack of supporting infrastructures, ineffective professional development, and lack of supporting technical support as barriers to digital technology use in vocational schools^14,16^.

### Needs for an instrument

When creating instruments, researchers include a sufficient set of appropriate indicators. The idea is to capture the most important feature of the structures. This study aimed to develop and validate a scale to model factors affecting digital technology access for instructional use in Indonesian vocational schools. Prior studies referred to the instrument development and validation of technology integration, resulting in some academic models. Technological pedagogical and content knowledge, or TPACK^19^, technology acceptance model, or TAM^20^, and theory of planned behavior, or TPB^21^ are examples of the models. These models have been adapted and tested in different contexts and settings^22,23^. Similar to the prior studies^4–6^, which explored the instrumentation processes for the digital divide, the current study also addresses a similar topic with a different context and setting, vocational school teachers in Indonesia.

### Correlations; digital divide variables

Studies on correlations regarding the digital divide framework have been conducted^6,12,24–26^. For example, Wei et al. (2011) presented the intercorrelation of adapted van Dijk’s three-level digital access model^12^. They created a model with three hierarchical tiers of factors: (1) digital access divides, (2) digital capacity divides, and (3) digital outcome divides. The findings revealed a link between the variables. For instance, individuals with no computers at home were shown to possess modest self-efficacy despite having access to enabling technology resources in the classroom. They also informed unsatisfying learning results among the students^12^. The origin of implementing and quantifying the digital divide is another example demonstrating the existence of connections among technology integration availability^6,24–26^. Barzilai-Nahon et al. (2006) discovered a correlation between many aspects of the digital divide, such as respondents’ demographics, accessibility, utilization, facility, context, and assistance^24^. Accessibility or material access not only has a direct impact on the digital divide but also has an indirect impact on use access. The connection approaches were used to determine causal intercorrelation among digital divide issues^2^.

### Model and hypotheses

**Figure 1:**
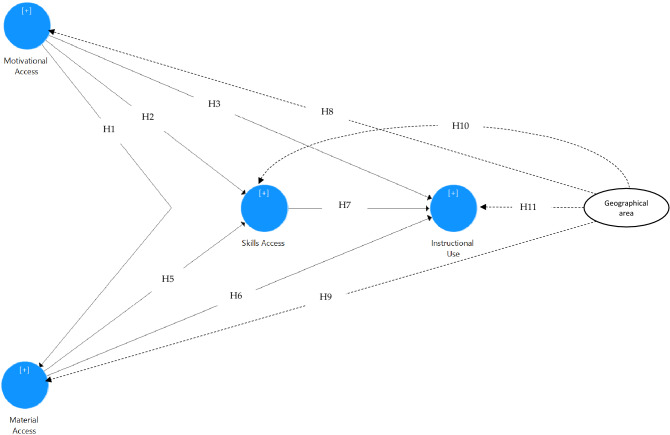
Proposed model.

In this study, we proposed a model comprising six hypotheses of the structural model and four hypotheses of differences. Figure 1 exhibits the proposed model of the study. The proposed model and scale refer to the context and setting of the digital divide perceived by vocational school teachers in Indonesia, adding to the geographical differences.

### Motivational access

Motivational access in this study is defined as vocational school teachers’ readiness to incorporate digital technologies during their teaching. Technology integration in teaching needs teachers’ readiness^27^. Based on van Dijk’s theory, two types of motivation access were proposed: external and internal motivation. Commitment to incorporating digital technology into educational activities to achieve specified learning objectives is external motivation. Meanwhile, internal motivation is a dedication to teaching with technology motivated by personal preferences and necessaries^28–30^. Regarding the motivational access, three hypotheses (H1, H2, and H3) were proposed.

H1. Motivational access significantly predicts material access.

H2. Motivational access significantly affects skills access.

H3. Motivational access is a significant predictor of instructional use.

### Material access

Categorical inequalities in society lead to an unequal allocation of resources, leading to unequal access to digital technology, known as material access^2,3^. Social and technological settings have an impact on the appropriation process. Personal and positional differences among users create the social context. Variations in technology access caused by resources perpetuate inequalities of involvement, resulting in increased inequalities between people, positions, and resources. Economic resources, specifically the income required to buy and maintain digital technology, are likely to significantly impact material access. In comparison to people with low income, people with high income have more desktops, laptops, and consoles. Therefore, this study proposed that material access has a major impact on skill access and instructional usage. The categorical inequalities may affect the users’ skills in educational activities, especially during teaching. In a recent report^30^, material access was significant in predicting skills access and use. Two hypotheses were proposed for the role of material access on skill access and instructional use.

H4. Material access significantly affects skills access.

H5. Material access positively influences instructional use.

### Skills access

Teachers’ capacity to use, connect, control, and grasp digital technology is skills access^30^. Three skills are included in the determined phases of digital technology access: strategic, informational, and operational skills. In this study, vocational school teachers’ strategic skills are their abilities to use digital technologies. The capacity to manage digital technology, such as smartphones, laptops, and the Internet, is classified as operational skills^2,6^. Informational skills refer to vocational school teachers’ capacity to find, choose, and interpret information using digital technologies, particularly the Internet and data-sharing technologies^5,6,30,31^. One hypothesis was established to report the effect of skills access on instructional use perceived by Indonesian vocational school teachers;

H6. Skills access has a positive effect on instructional use.

### Instructional use

Within the context of this study, the phrase “instructional use” aimed to denote (van Dijk, 2005) usage of access to digital technology. It is the result of integrating the outcomes of motivation, material, and skills access^3^. Within the context of this research, the phrase itself can be explored in terms of how vocational school teachers use digital technology in their classrooms^5,6^.

### Geographical area

The expansion of digital technology performs an increasingly vital role in economic, social, geopolitical, and social settings. Even though digital technology has reached nearly every part of the planet, a digital divide stems from geographical differences between urban and rural areas^32,33^. Oyelaran-Oyeyinka and Lal (2005) state that poor online activity is frequently caused by a lack of infrastructure and low ownership of computers and other technological devices^33,34^. According to Lesame and Robinson et al. (2015), education, income, and economic development inequalities between urban and rural areas are among the variables that impede technology integration^33,35^. Besides the structural model, the current study also elaborated on the differences between the suburban and urban locations regarding all proposed variables. In this study, urban areas relate to places with a large population. The term “urban” refers to the main metropolis and adjacent towns. On the other hand, suburban areas refer to residential regions (also known as suburbs). A suburb refers to the residential areas surrounding a larger city. They can be a part of a larger metropolis or a collection of residential communities spread out across a large area. Four hypotheses were proposed to meet the purpose of the study.

H7. A significant difference emerges for motivational access based on geographical area.

H8. A significant difference exists in material access based on geographical area.

H9. A difference in skills access is reported based on geographical area.

H10. There is a significant difference in instructional use based on geographical area.

## Method

The data used in this study was gathered using a survey. We developed the survey instrument by analyzing prior studies^36^. Afterward, the instrument was content-validated before being disseminated for a pilot study^37,38^. PLS-SEM was used to evaluate the model. We assessed the study model for causality using a predictive approach since the data distribution constraint did not hamper the process. In addition, t-tests were used to determine the difference among all involved constructs based on geographical areas, big and small cities.

### Instrument establishment

Researchers can use literature review to help them investigate a theoretical framework, choose relevant methodologies, and provide tools. We developed measures from prior studies^2,6,39^, resulting 30 items with four variables (motivational access, skills access, material access, and instructional access). Content validity was conducted through discussions with five experts in educational technology and policy. The procedure was carried out in the form of interactive dialogue. Some items were amended; two were removed because they did not suit the Indonesian context. This process creates a significant contribution to social, cultural, and setting suitability^40^.

We emailed 15 experts to assess the scale (28 items) for their relevance and clarity; ten agreed to participate using the content validity index (CVI)^41^. However, three experts refused to participate; two others had no responses. Item level (I-CVI) and scale level (S-CVI) were evaluated. The item was calculated by dividing the expert numbers giving a score of three or four (positive). I-CVI scores should not be less than 0.780 for the ten experts. S-CVI was calculated when the sum of the I-CVI by the item’s total number to determine the scale level was divided. Excellent content validity is represented by S-CVI/UA 0.800 and S-CVI/Ave 0.90. Using Microsoft Excel, we calculated the CVI scoring requirements. Two items were dropped due to the low value of I-CVI. The results were satisfactory after the elimination process, and the instrumentation scale’s validity (n-26) was verified in the initial stage of the instrumentation.

### Pilot study

The instrument was administered to 77 respondents for a pilot study after the I-CVI and S-CVI computations. The pilot study is essential to test a technique’s reliability in a small cohort before applying it to a larger-scale data collection^42–44^. A pilot study is required to investigate a novel intervention. The pilot study within the current context was evaluated through a reliability test^45^. We used the Statistical Package for the Social Sciences (SPSS) 25 to perform the reliability test. The findings were adequate to back up the scale’s reliability; no variable had a Cronbach’s alpha of < 0.700. Following the procedure, 26 items were addressed for the main survey.

### Data collection and preparation

The study population is all vocational teachers in Indonesia (315,553 teachers). This study’s sampling was governed by the ten-times rule, which required ten cases for a measured variable^46^. The minimum number of respondents (sample) for this study should be more than 60 because of the six lines of the structural model. However, we managed to obtain more data through the online survey. The data gathering took three months. Informed consent was obtained from all respondents. The need for ethics approval was waived by the IRB of Universitas Jambi. The study is in accordance with relevant guidelines and regulations.

All data (n. 1458) were computed in Microsoft Excel, and SPSS 25 was used to check for data normality. However, 103 responses were excluded due to the inaccuracy of the data. Finally, 1355 data were measurable and included in the analysis, 551 were males, and 804 were females. From the data, 1063 respondents had five years of teaching experience, while the others (n. 292) had five years or less of teaching experience. Further, 824 respondents lived and worked in suburban areas; 531 respondents were in urban areas.

### Data analysis

The structural equation modeling method (SEM) was used to analyze the data quantitatively. The partial least square SEM (PLS-SEM) was used, which provides more reliable structural model estimations than covariance-based SEM (CB-SEM)^47^. The method is a strong multivariate statistical technique that combines factor analysis and multiple regression to investigate the structural links between a set of measurable and latent variables. Understanding the pattern and degree of correlations/covariances between variables and adjusting for variance are two main goals of SEM. The findings are susceptible to missing data, outliers, and sample size, similar to the standard statistical methods. SEM has been a popular tool in a variety of applications and fields of study, namely economics^48^, education^49,50^, finance^51^, and healthcare^52^. SEM consists of two types of latent components: endogenous and exogenous constructs. Exogenous constructs are independent variables, whereas endogenous constructs are dependent variables. PLS-SEM procedures recommend two assessment phases, measurement and structural. Data preparation and descriptive statistics are presented before presenting the two phases. Path coefficients (β), t-value, p-value, coefficient of determination (*R*^2^), and the effect size of (*f*^2^) were assessed to elaborate the relationships between variables^53^. In addition, the t-test was addressed in the SPSS on geographical areas regarding all four variables: instructional use, material access, motivational access, and skills access.

## Findings

### Data preparation and descriptive statistics

The current study used SPSS 25 program to address data screening issues; missing data, multicollinearity, outlier detection, and normality. The box plot for each sub-construct was used to identify outliers^54,55^; no outliers issues emerged. The criteria for univariate normality were measured by calculating the skewness and kurtosis values (1.96 to + 1.96)^56^. The percentage of missing data ranged from 0 to 0.5% for every item. The data that was missing was fully random. Table 1 shows all variables’ mean, standard deviations, skewness, and kurtosis. The skewness and kurtosis scores ranged from −0.828 to 1.429. Thus, the data were normally distributed. All variables obtain satisfactory results of the mean. The statistical computation shows that instructional use had the highest mean (M = 4.019) while material access gained the lowest (M = 3.682).

**Table 1:** Mean, standard deviation (SD), skewness, and kurtosis.

Variable	Mean	SD	Skewness	Kurtosis
Motivational access	3.912	0.696	−0.828	1.429
Skills access	3.870	0.618	−0.569	0.948
Material access	3.682	0.805	−0.345	0.185
Instructional use	4.019	0.687	−0.511	0.403

### Measurement model

**Figure 2:**
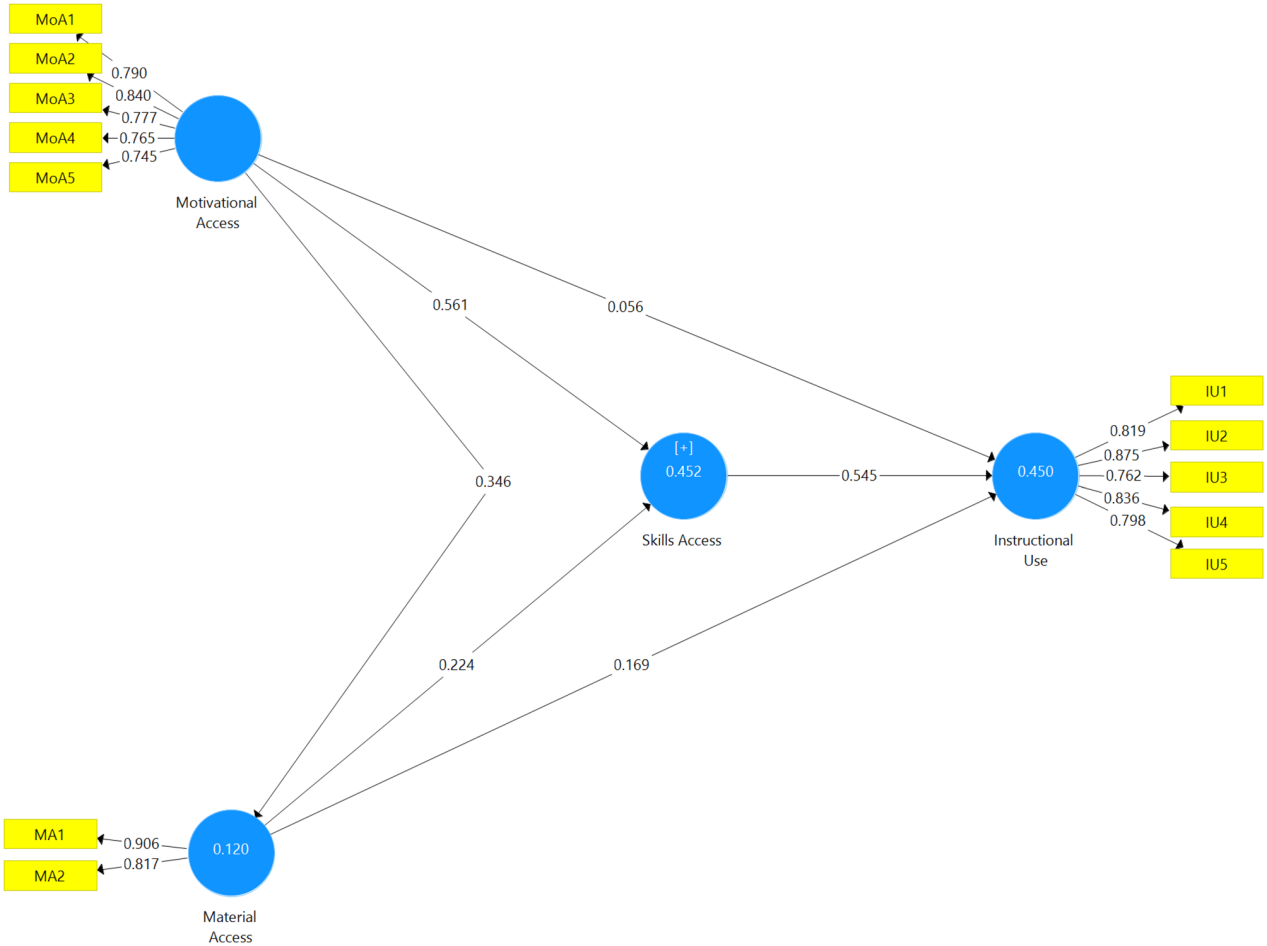
Measurement model.

The measurement model is used to report the model’s reliability and validity. Reflective indicator loadings, internal consistency reliability (ICR), convergent validity, and discriminant validity are the four stages of reflective measurement models^57^. Examining the indicator loadings is the first stage in evaluating the reflective measurement model. A loading value that is greater than 0.708 is recommended^58^. The criterion refers to constructions that account for more than 70% of the variation, making the items more reliable. However, the retainment of a value of 0.500 was also suggested^59,60^. We generated the data using SmartPLS 3.3.3 to display the loadings of all items. Four indicators with loadings of < 0.500 (MoA6, MoA7, SA1, and SA2) were eliminated. The elimination procedure aimed to retain the proposed model’s validity and reliability^61^. Table 2 and Fig. 2 comprehensively summarize the loadings (22 items). Skills access (SA5; 0.697) offered the lowest value, whereas material access yielded the highest (MA2; 0.906).

**Table 2:** Factor loading, reliability, and validity of measurement model.

Construct	Items	Load	α	rho_A	CR	AVE	VIF
Instructional use	IU1	0.819	0.877	0.881	0.910	0.670	2.263
	IU2	0.875					2.748
	IU3	0.762					2.043
	IU4	0.836					2.436
	IU5	0.798					1.929
Material access	MA1	0.906	0.663	0.705	0.853	0.744	1.326
	MA2	0.817					1.326
Motivational access	MoA1	0.790	0.843	0.843	0.888	0.615	1.999
	MoA2	0.840					2.362
	MoA3	0.777					1.749
	MoA4	0.765					1.656
	MoA5	0.745					1.568
Skills access	SA10	0.775	0.908	0.911	0.924	0.548	2.269
	SA11	0.777					2.531
	SA12	0.721					2.180
	SA3	0.706					1.877
	SA4	0.708					2.263
	SA5	0.697					2.116
	SA6	0.710					2.021
	SA7	0.759					2.151
	SA8	0.742					2.078
	SA9	0.801					2.479

### ICR

For the ICR, Rho_A, Cronbach’s alpha, and Composite reliability (CR) were statistically verified through the computation process in the SmartPLS^62^. Rho_A with greater values indicates higher levels of reliability. Satisfactory values are those that exceed 0.700. Values of 0.950 and above, on the other hand, are problematic because they can be defined as a sign of lowering construct validity. Items with values of 0.950 and higher are more likely redundant^58,62,63^. Other measurements of internal consistency dependability include Cronbach’s alpha and CR. Cronbach’s alpha ($\mathrm{\alpha })$ values should also be greater than 0.700. The exact values of rho_A, Cronbach’s alpha, and CR were exhibited in Table 2. All constructs had good rho_A, Cronbach’s alpha, and CR values, indicating well-established ICR.

### Convergent validity

The degree (construct converging to explain item variance) is defined as convergent validity. We applied the average variance extraction (AVE) to compute convergent validity. In the SmartPLS, each loading is squared on a variable. The minimum AVE should be 0.500, explaining 50% or more of the variance. The PLS-SEM procedure produces AVE values greater than 0.500 (see Table 2). The variable “skills access” was revealed to have the lowest value of AVE (0.548), elaborating the variance (54%). Material access obtains the highest AVE of 0.744, accounting for a variance of 74%. Thus, AVE values support the convergent validity.

### Discriminant validity

The amount to which a variable differs empirically from other variables is known as discriminant validity. Cross-loading and the heterotrait-monotrait (HTMT) ratio were used to determine discriminant validity^64,65^. The discriminant validity exists in the absence of cross-loading; a variable’s loading is greater than the sum of its cross-loadings. From the computation results, each loading in a variable is higher than all cross-loadings on the other variables (Table 3). For instance, IU2, as one of the items of instructional use, has the highest loading of 0.875 (material access 0.387, motivational access 0.417, and skills access 0.555). HTMT values should be lower than 0.900 for the discriminant validity to exist. Once the HTMT value exceeds 0.900, the variables share similar ideas^66^. All HTMT values in Table 4 gain values of less than 0.900, indicating the values differ from 1. The discriminant validity of the current study was demonstrated based on the evaluation of cross-loading and HTMT.

**Table 3:** Cross-loading.

	Instructional use	Material access	Motivational access	Skills access
IU1	**0.819**	0.391	0.410	0.528
IU2	**0.875**	0.387	0.417	0.555
IU3	**0.762**	0.231	0.321	0.476
IU4	**0.836**	0.321	0.387	0.571
IU5	**0.798**	0.358	0.349	0.528
MA1	0.403	**0.906**	0.337	0.414
MA2	0.303	**0.817**	0.252	0.293
MoA1	0.329	0.244	**0.790**	0.491
MoA2	0.365	0.263	**0.840**	0.547
MoA3	0.355	0.267	**0.777**	0.489
MoA4	0.368	0.276	**0.765**	0.490
MoA5	0.391	0.304	**0.745**	0.483
SA10	0.551	0.382	0.506	**0.775**
SA11	0.525	0.275	0.547	**0.777**
SA12	0.520	0.269	0.511	**0.721**
SA3	0.403	0.250	0.471	**0.706**
SA4	0.443	0.268	0.466	**0.708**
SA5	0.412	0.271	0.462	**0.697**
SA6	0.456	0.346	0.390	**0.710**
SA7	0.470	0.326	0.443	**0.759**
SA8	0.488	0.396	0.395	**0.742**
SA9	0.524	0.309	0.519	**0.801**

Significant values are in bold.

**Table 4:** HTMT ratio and model fit.

	Instructional use	Material access	Motivational access	Model fit	
Instructional use				SRMR	0.062
Material access	0.532			d_ULS	0.958
Motivational access	0.535	0.455		d_G	0.369
Skills access	0.724	0.527	0.727		

### Structural model

Before reporting the structural model of the study, we elaborated the model fit for a better presentation of the proposed model evaluation^67^. Three criteria of fit indices were determined in this study; standardized root mean square residual (SRMR), d_ULS, and d_G. The SRMR was mainly used to measure the fit; it is the differentiation between the noticed relationship and the model for the correlation matrix^68^. It is a quantitative indicator that evaluates how well a model fits the data by measuring the average size of the discrepancies between observed and expected correlations^68^. The threshold of SRMR is less than 0.08. The criteria d_ULS and d_G were used to be another reference for the fit assessment; there are no cut-off values for the d_ULS and d_G. Table 4 shows the sufficient values of the measurement for the model fit, SRMR of 0.062, d_ULS of 0.985, and d_G of 0.396.

To assess the structural model, the data were bootstrapped (5000-subsamples). Only one hypothesis is reported to be insignificant (H3). The structural model of the other four hypotheses (H1, H2, H4, H5, and H6) are informed significant, assuming a 5% significance level (Table 5). The findings of the study support H1; motivational access significantly affects material access (β = 0.3460; t = 11.8690; p < 0.01). Hypothesis 2 that examines the relationship between motivational access and skills access is also supported (β = 0.5610; t = 23.8650; p < 0.01). On the other hand, the insignificant predicting power emerges on the role of motivational access to instructional access (β = 0.5610; t = 23.8650; p < 0.01), opposing hypothesis three. For hypothesis 5, material access is reported to significantly predict skills access (β = 0.2240; t = 9.1810; p < 0.01). The significant relationships are also linked between material access and instructional use (β = 0.1690; t = 6.6840; p < 0.01) as well as between skills access and instructional use (β = 0.5450; t = 17.0450; p < 0.01).

**Table 5:** significant tests and effect sizes (*f*^2^).

H	Path	Β	t-value	p-values	Sig	*f*^2^
H1	Motivational access → material access	0.3460	11.8690	0.0000	Yes	0.136
H2	Motivational access → skills access	0.5610	23.8650	0.0000	Yes	0.506
H3	Motivational access → instructional use	0.0560	1.5750	0.1150	No	0.003
H4	Material access → skills access	0.2240	9.1810	0.0000	Yes	0.080
H5	Material access → instructional use	0.1690	6.6840	0.0000	Yes	0.042
H6	Skills access → instructional use	0.5450	17.0450	0.0000	Yes	0.296

**Figure 3:**
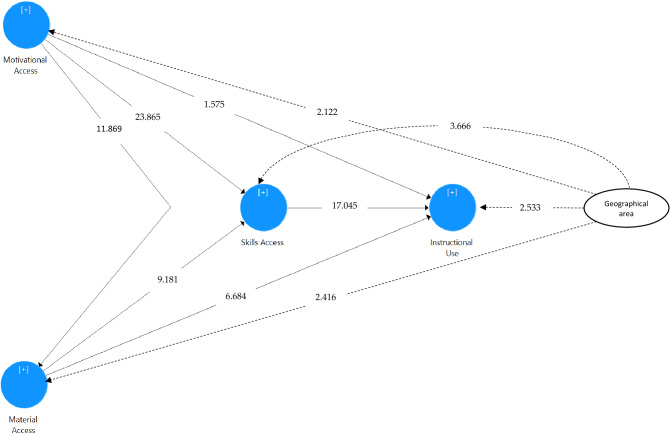
Structural model (t-value).

Prior researchers have recommended the coefficient recommendation (*R*^*2*^) as a measure to assess the structural model^69–71^. *R*^2^ is a statistical definition when a value measures the predictive accuracy, calculated as the correlation of squares between a certain dependent variable^71–73^. The *R*^2^ value is calculated, ranging from 0 to 1. A more robust value of R^2^ has an indication of greater predictive accuracy. The *R*^2^ value of 0.25 is defined as weak; 0.75 (substantial); 0.50 (moderate). The computation shows that all model-dependent variables obtained good levels of *R*^2^. The value of material access is 0.120 (weak), skills access is 0.452 (moderate), and instructional use is 0.450 (moderate) (Fig. 3).

The effect size symbolized with *f*^2^^74^ is a measurement of the size of an effect, independent of the size of the sample. The primary measures of *f*^2^ are commonly used in PLS-SEM. The commonly applied in PLS-SEM is Cohen’s *f*^2^ coefficient^74^ (Cohen, 1992), computed as ∆*R*^2^⁄ (1 – *R*^2^). ∆*R*^2^ is the gradual input of a predictor latent variable to the *R*^2^ of the criterion latent variable to which it points. The other measurement in PLS-SEM is the complete input of the predictor latent variable; the numerator ∆*R*^2^ of Cohen’s *f*^2^ equation^74^. However, the later measurement produces lower computations, a more conservative *f*^2^. By convention, *f*^2^ value of 0.02, 0.15, and 0.35 is termed small, medium, and large. The computation shows that all exogenous have effect sizes to endogenous variables presented in Table 5. Motivational access gains an *f*^2^ value of 0.136 (medium) on material access; motivational access to skills access (0.506, large), motivational access to instructional use (0.003, small), material Access to skills access (0.080, small), material access to instructional use (0.042, small), and skills access to instructional use (0.296, large).

### Differences based on geographical areas

Besides the structural model, the difference test was computed regarding the geographical areas of the vocational schools we surveyed, suburban and urban. We focused on the geographical areas since this demographic characteristic gives what to plan for all related stakeholders in vocational education and what to invest for digital technology use in suburban and urban areas. Using the t-test, the differences were calculated. Significant differences emerge in all variables regarding the geographical areas (Table 6). For motivational access, the difference is the least significant, with a t-value of 2.112 and a p-value of less than 0.05; the mean difference (MD) is −0.08175. The perception of respondents from urban areas is slightly higher than those from suburban areas. The most significant result appears in the mean difference in skills access. The respondents from suburban perceived the variable much higher than their counterparts from urban areas of schools (t = 3.666; p < 0.001) with a mean difference of −0.12542.

**Table 6:** Significant differences of all variables regarding geographical areas, suburban and urban.

	Areas	n	Mean	t-value	p-value	MD	Sig
Motivational access	Suburban	824	3.8795	2.112	0.035	−0.08175	Yes
	Urban	531	3.9613				
Skills access	Suburban	824	3.8204	3.666	0.000	−0.12542	Yes
	Urban	531	3.9459				
Material access	Suburban	824	3.6396	2.416	0.016	−0.10808	Yes
	Urban	531	3.7476				
Instructional use	Suburban	824	3.9813	2.533	0.011	−0.09666	Yes
	Urban	531	4.0780				

## Discussions

The scale development for the current study was addressed within some procedures that aim at establishing a valid and reliable model. We analyzed prior academic sources and successfully adapted 30 items. We validated the scale using content validity (discussion with experts) and CVI. Afterward, it was piloted and computed for Cronbach’s alpha. The results of the two statical processes present four items to be dropped. Twenty-six indicators for the main data collection were addressed. The cleaned data, 1355 responses, were assessed for the measurement model. Four items were dropped in this phase; Finally, 22 items were distributed for the main data collection, structural model, and tests of difference. Prior studies have also presented similar procedures for their valid and reliable scales^6,75,76^. Reporting valid and reliable scales in a social quantitative study is important for specific contexts and settings.

For the structural model, five statistically significant relationships are reported. One hypothesis, nevertheless, is ruled out. Motivational access is shown to be a significant predictor of material access (H1). The intention of teachers to integrate digital technologies into instructional activities has an impact on resource disparities for unequal access to digital technology, particularly the Internet, as perceived by vocational school teachers. Similar findings were found by prior research^28–30^. Motivational access is also important in predicting skills access (H2), which confirms the outside and inside commitment to using digital technology to facilitate skills access and the capacity to use technology to advocate their stance to students. Similar findings have been observed from earlier studies on these correlations^6,24–26^. However, motivating access does not significantly predict access to digital technology for the purposes of teaching and learning (H3). It is clear that Indonesian vocational school teachers’ inner and outer commitment to using digital technology in the classroom has no influence on technology integration for instructional activities. The result argues previous research that found a substantial link between motivational access and technological use.^25,27,30^.

Material access is described as variances in Internet access generated by resources that maintain social inequalities, leading to greater inequalities between persons, jobs, and capabilities. Both skills access and instructional use are significantly predicted by material access (H4 & H5). Van Deursen and van Dijk (2005)^30^ revealed that material access was significant in predicting both skill access and usage, which is comparable to the findings of this study. It demonstrates that categorical disparities have a contribution to unbalance allocation of resources, resulting in disproportionate access to vocational school teachers’ digital aptitude.

Skills access is a strong predictor of instructional use; how Indonesian vocational school teachers utilize digital technology in teaching (H6). Van Dijk drew a systematic line of linkages in his initial theory of digital technology access. The skills access arrow was enlightened to correspond with the use of technology; the theory established the correlation of the technology access notion^2^. The new conclusion confirms prior findings, indicating that technological skills have a substantial impact on how it is used during instructional activities^30,31,77^. The results support the idea that digital technology skill availability is a fundamental factor in technology integration and access in education.

Furthermore, the current research investigated the significant differences in geographical areas based on all variables involved. All the hypotheses are accepted (H7, H8, H9, H10). The results could be related to the disparity in digital technology access to facilities and skills between urban and suburban vocational school teachers. Although digital technology has reached almost every corner of the globe, there is a digital divide due to geographical inequalities in technological infrastructure and innovative change activities between towns and cities^32,33^. Oyelaran-Oyeyinka and Lal (2005) informed that lack of infrastructure and limited ownership of computers and other technological gadgets are frequently the causes of poor internet engagement^34^. According to Lesame (2013) and Robinson et al. (2015), education, income, and economic development inequalities between urban and rural areas are among the variables that impede technology integration in education^33,35^.

## Conclusion

The present report’s valid and reliable measure has psychometric features to aid future researchers in capturing teachers’ access to digital technologies. Nevertheless, the reliability and validity of the instrument are only used to assess Indonesian vocational school teachers’ access to digital technology during teaching. Thus, studies in other settings and contexts should be done. Future research should include a larger sample size. More broadened features for the framework proposed in this study could aid in the development of more instruments. Furthermore, academics must create larger-scale definitions for the integration specifications, such as m-learning, social networking, and eLearning. Teachers’ perspectives, according to research, play a critical part in the success of new initiatives, as teachers will be the ones to lead the use of technology in the classroom. This research has shed light on the critical role that specific access plays in teachers’ usage of digital technology in the classroom in Indonesia.

While the outcomes of this study have several limitations, the methodologies used yielded substantial results. The findings are extremely useful in gaining a better understanding of educational access to digital technologies. The variation in digital technology utilization for education depending on demographic areas is also substantial, largely due to the experience of the teachers and the location of the schools. Related policymakers should prepare initiatives properly for professional development and activities as attempts to improve the use of digital technology in the classroom, particularly for teachers in suburban areas. In general, the outcomes of this study can be used as a guideline for the purpose. Other demographics, such as teachers’ gender, experience, and age are recommended to be included in future research (Supplementary Information).

## Supplementary Information

Supplementary Information.

## Data Availability

The datasets generated and analysed during the current study are available in the Figshare repository (https://doi.org/10.6084/m9.figshare.19880215.v1).
